# Supplementary material for: Gene Network Analysis of the Transcriptome Impact of SARS-CoV-2 Interacting MicroRNAs in COVID-19 Disease
Source: Int J Mol Sci. 2022 Aug 17;23(16):9239. doi: 10.3390/ijms23169239 (PMC9409149; doi:10.3390/ijms23169239)
Supplement: Supplementary file 1 [file ijms-23-09239-s001.zip › Supplementary file S5.pdf]

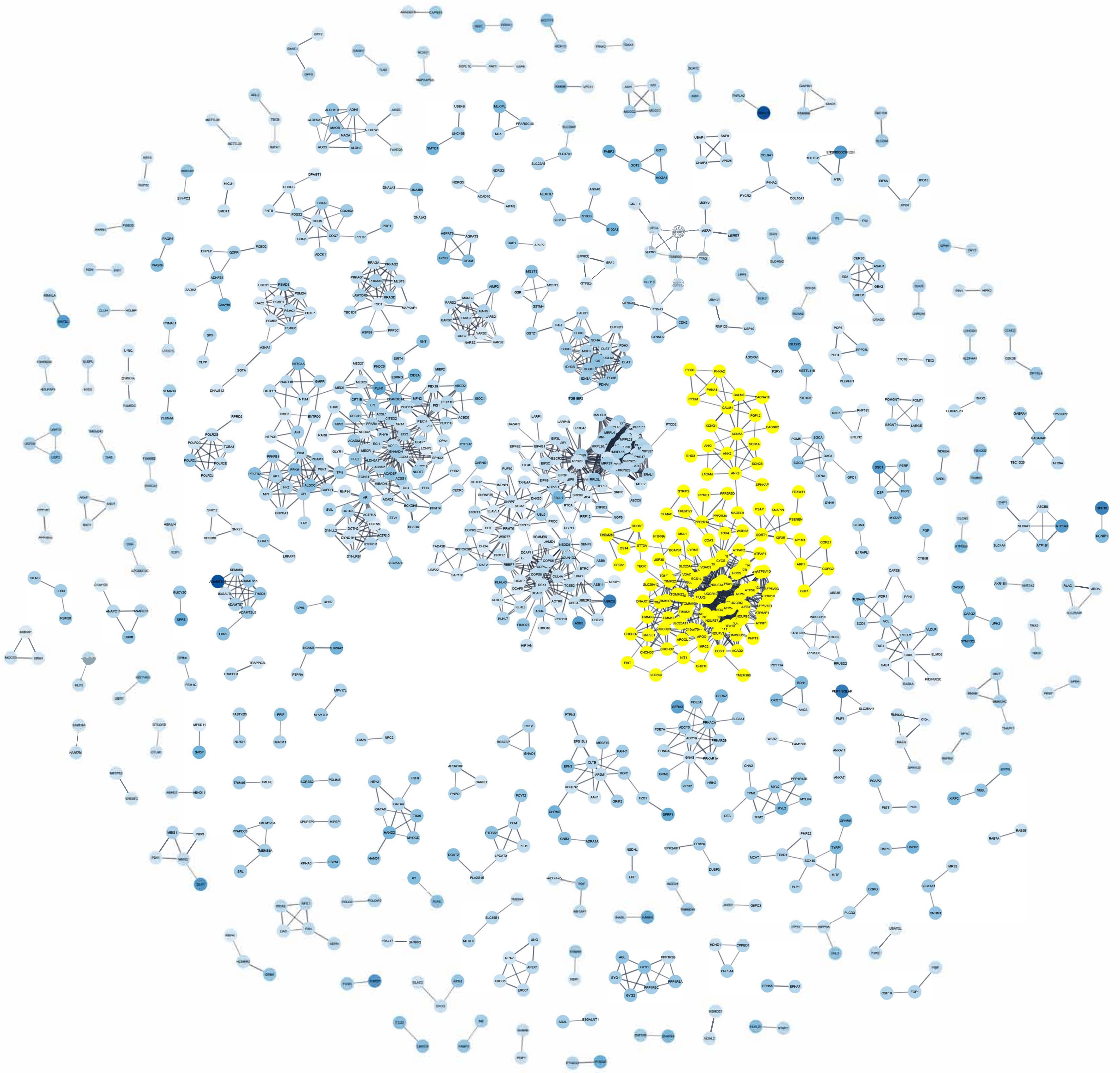

Figure S1. Clustered network of the down-regulated genes in heart, targeted by microRNA

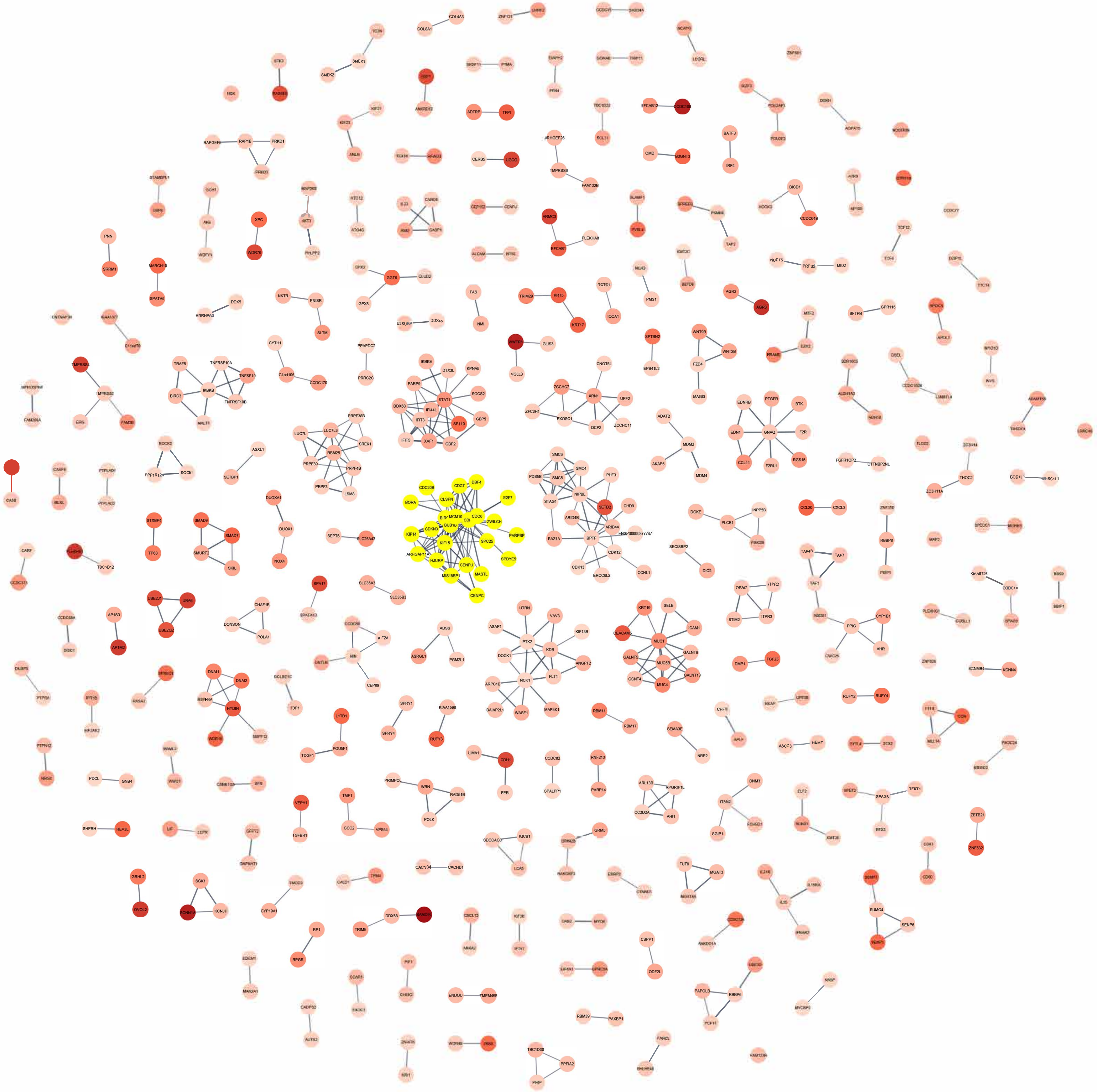

Figure S2. Clustered network of the up-regulated genes in heart, targeted by microRNA

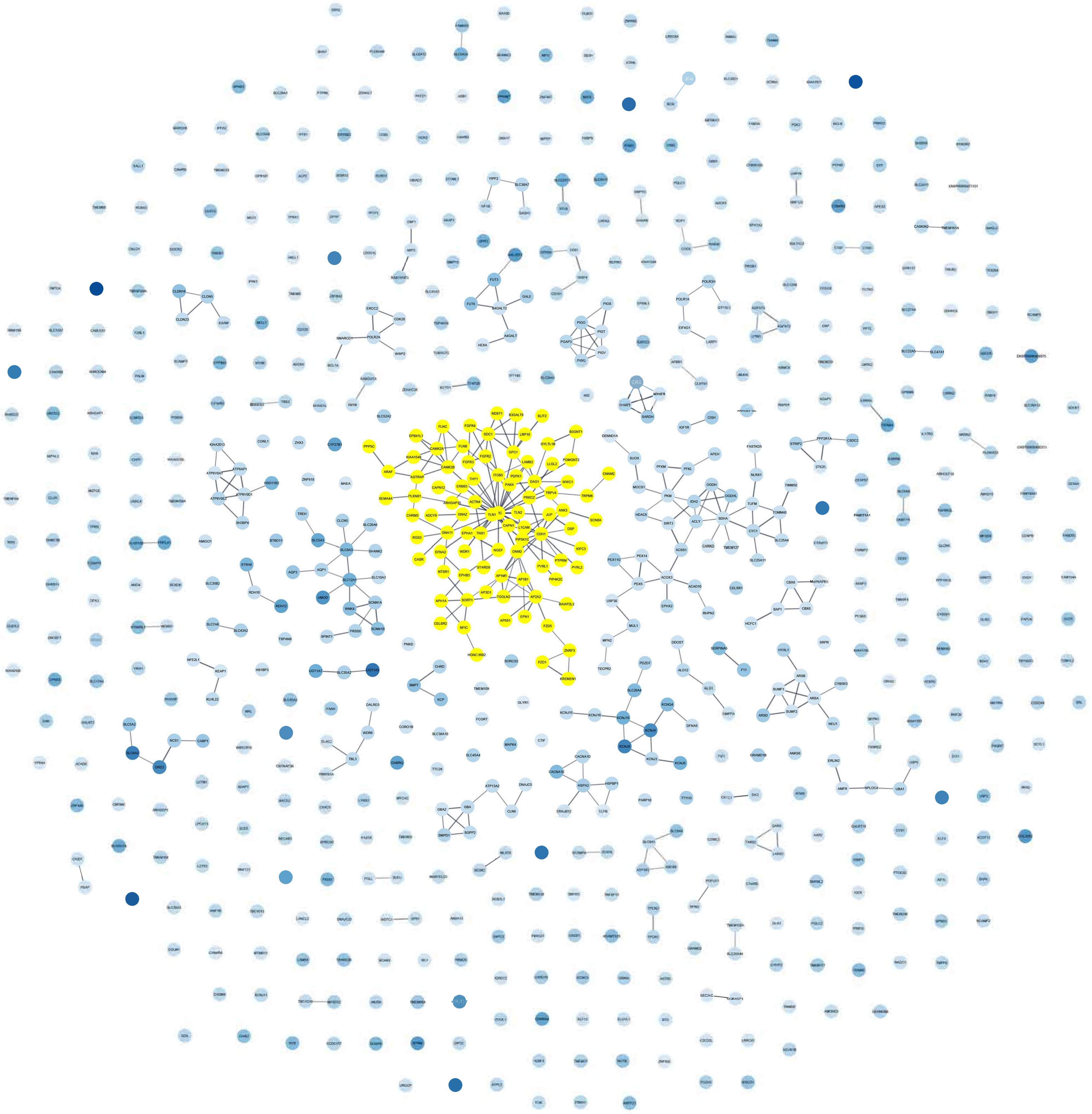

Figure S3. Clustered network of the down-regulated genes in kidney, targeted by microRNA

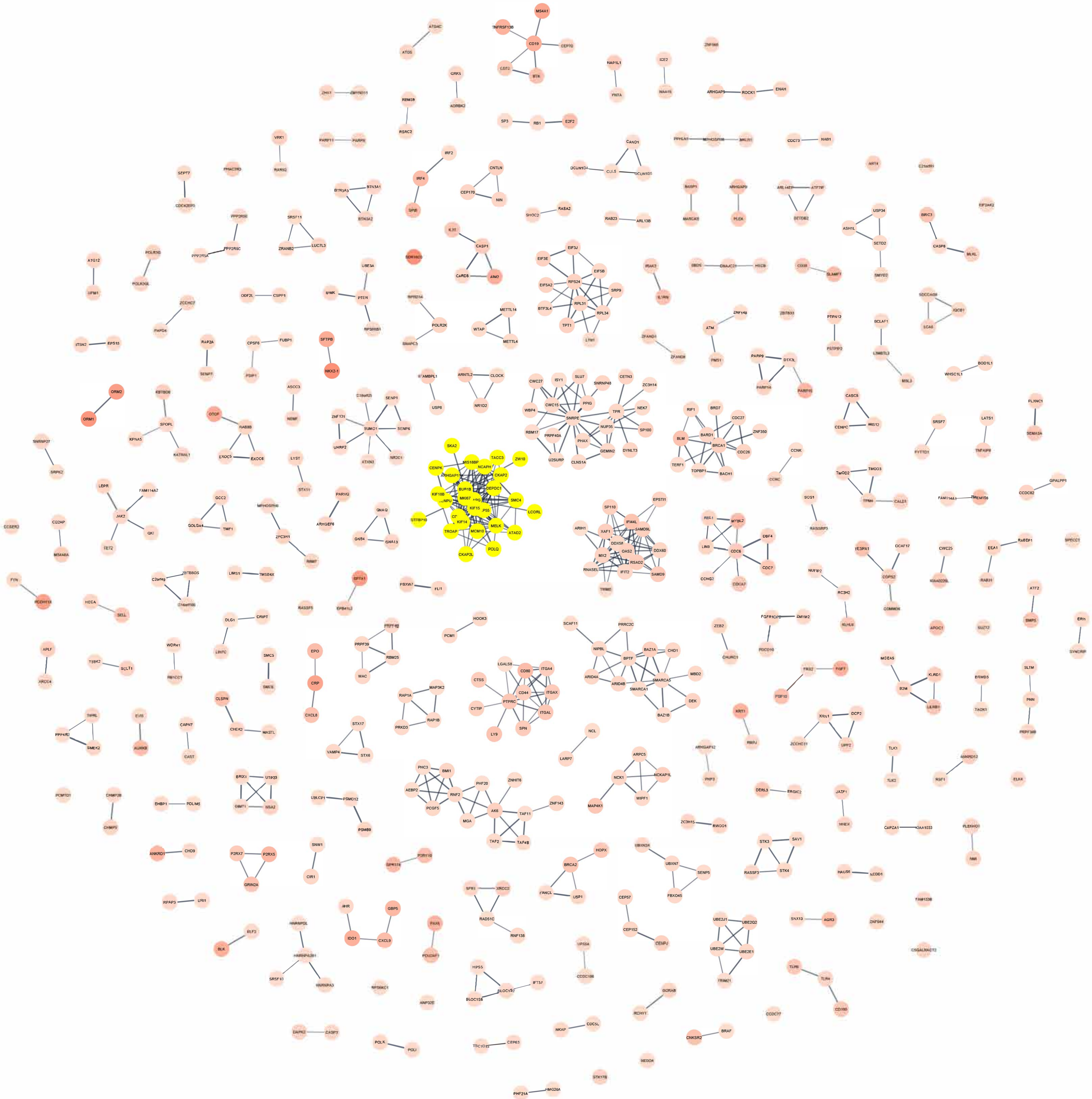

Figure S4. Clustered network of the up-regulated genes in kidney, targeted by microRNA

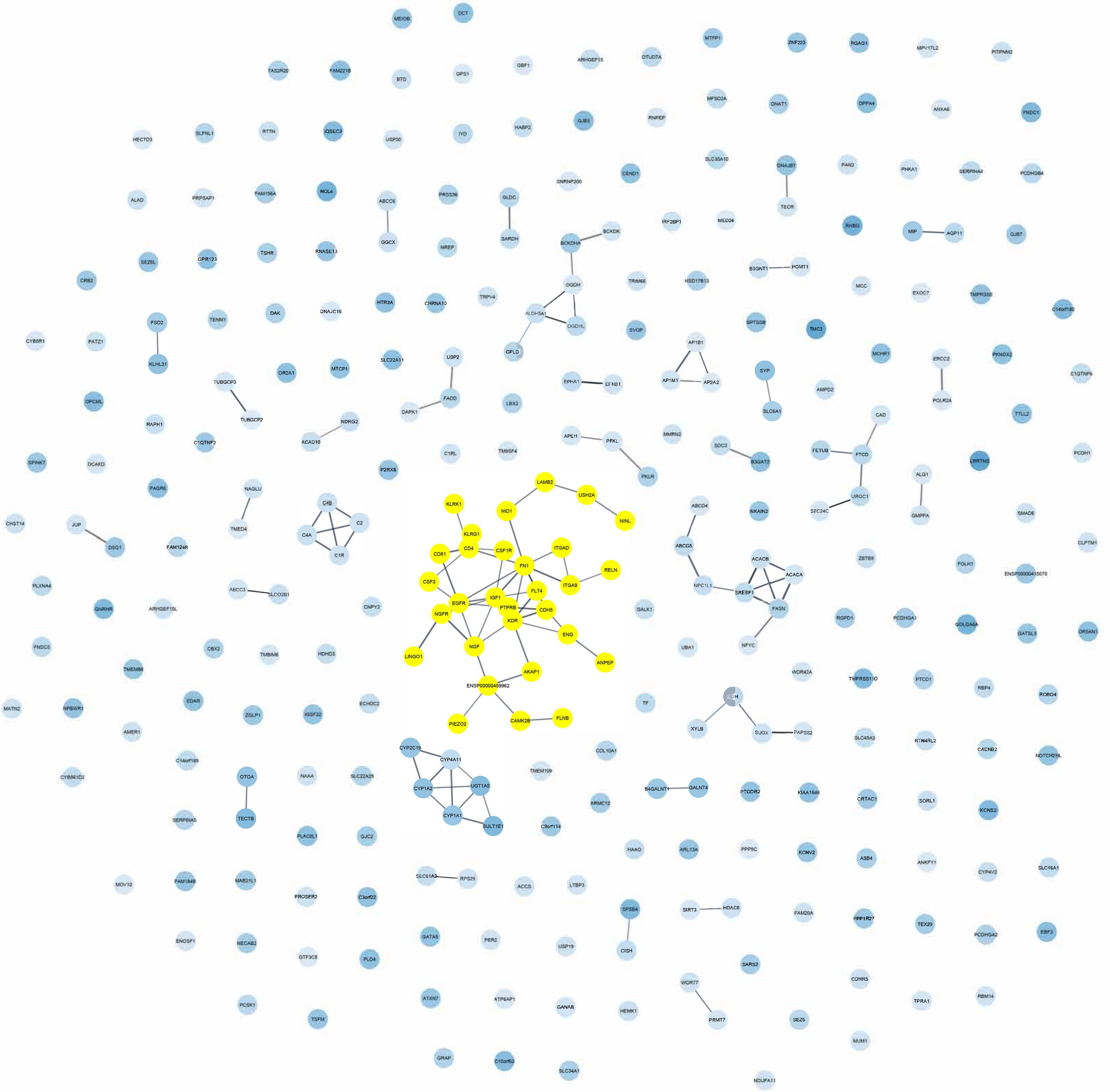

Figure S5. Clustered network of the down-regulated genes in liver, targeted by microRNA

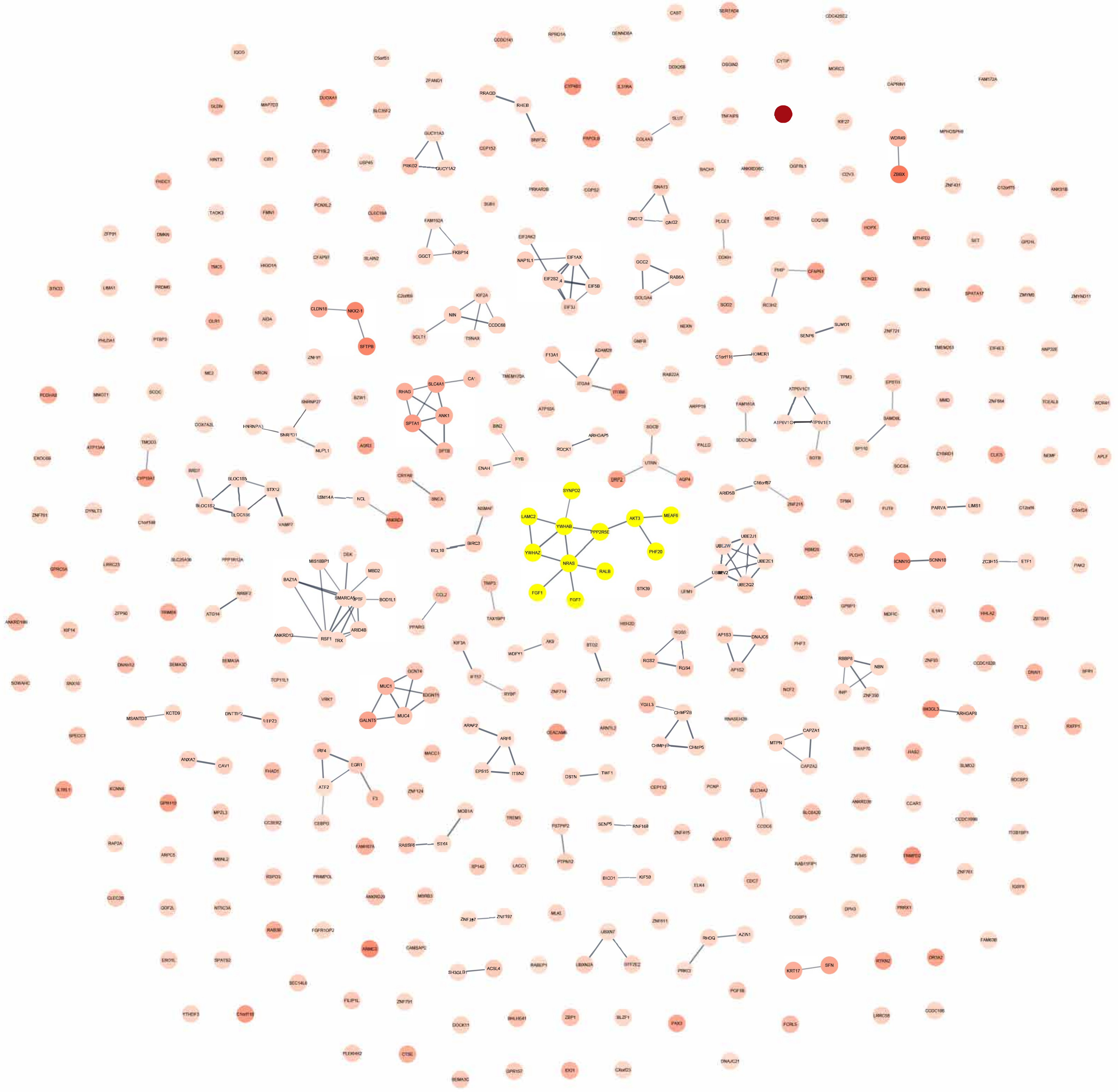

Figure S6. Clustered network of the up-regulated genes in liver, targeted by microRNA

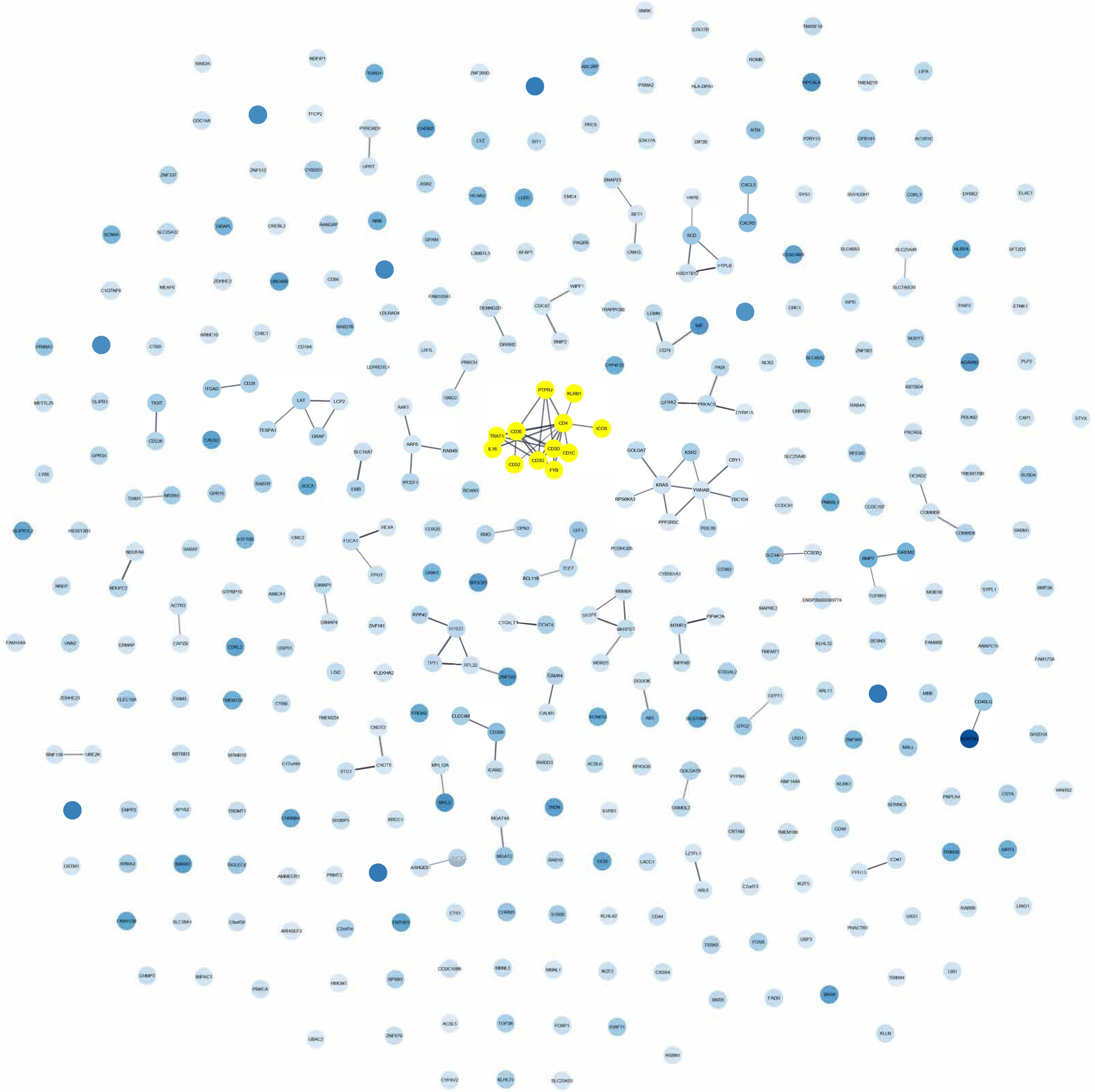

Figure S7. Clustered network of the down-regulated genes in lymph node, targeted by microRNA

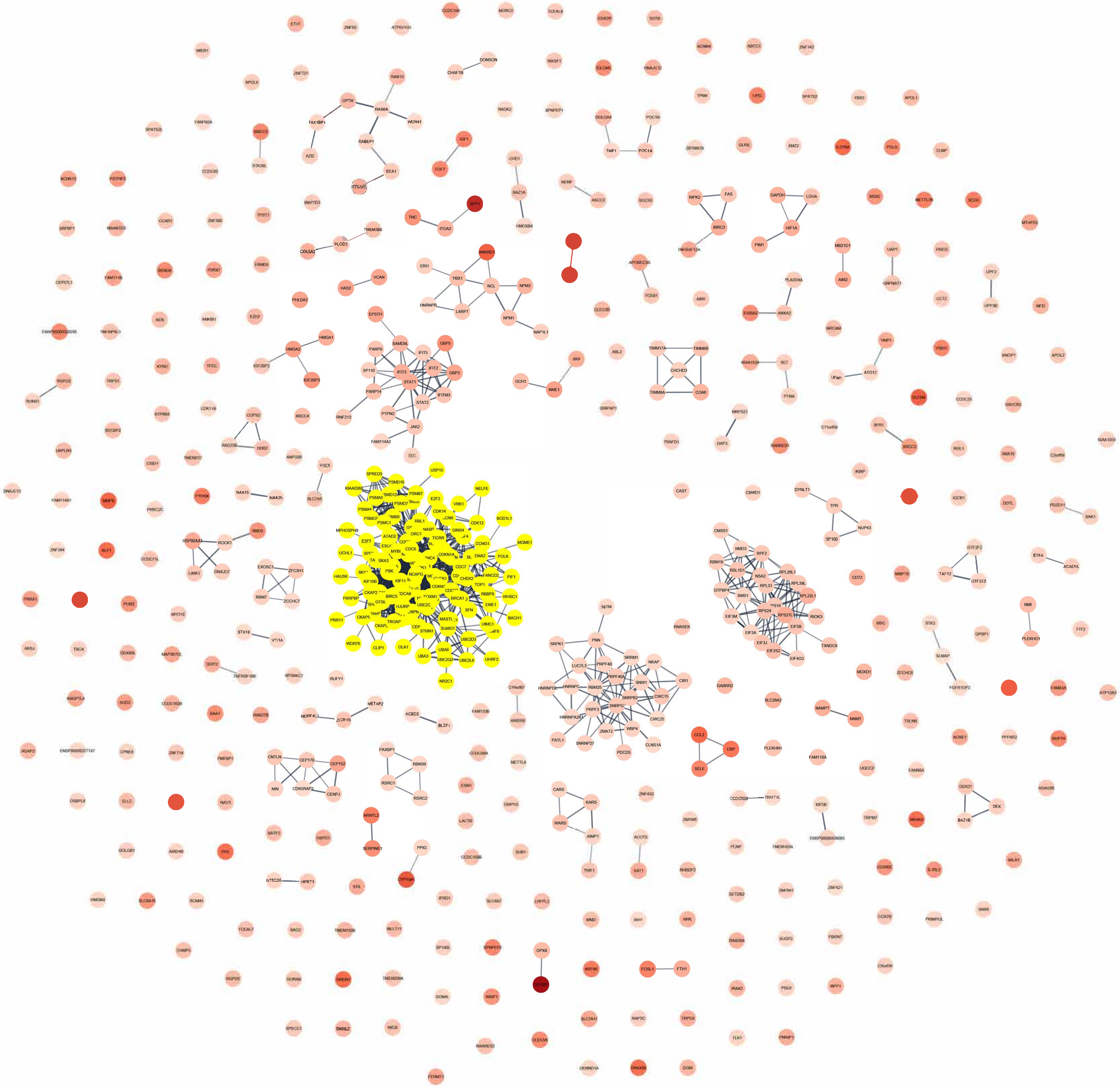

Figure S8. Clustered network of the up-regulated genes in lung, targeted by microRNA
